# Mastering your fellowship

**DOI:** 10.4102/safp.v62i1.5208

**Published:** 2020-09-07

**Authors:** Klaus B. von Pressentin, Mergan Naidoo, Bob Mash, Andrew Ross, Tasleem Ras

**Affiliations:** 1 Division of Family Medicine, University of Cape Town, Cape Town, South Africa; 2 Department of Family Medicine, University of KwaZulu-Natal, Durban, South Africa; 3 Department of Family and Emergency Medicine, Stellenbosch University, Cape Town, South Africa

**Keywords:** Fellowship of the College of Family Physicians of South Africa (FCFP [SA]) examination, family medicine registrars, registrars, residency, primary care, child health

## Abstract

The series, ‘Mastering your Fellowship’, provides examples of the question format encountered in the written and clinical examinations, Part A of the FCFP (SA) examination. The series is aimed at helping family medicine registrars (and their supervisors) prepare for this examination. Model answers are available online.

## Introduction

This article in the South African Family Practice journal is aimed at helping registrars prepare for the FCFP (SA) Final Part A examination (Fellowship of the College of Family Physicians) and will provide examples of the question formats encountered in the written examination: Multiple Choice Question (MCQ) in the form of Single Best Answer (SBA – Type A) and/or Extended Matching Question (EMQ – Type R); Short Answer Question (SAQ), questions based on the Critical Reading of a journal (evidence-based medicine) and an example of an Objectively Structured Clinical Examination (OSCE) question. Each of these question types are presented based on the College of Family Physicians blueprint and the key learning outcomes of the FCFP programme. The MCQs will be based on the 10 clinical domains of family medicine, the MEQs will be aligned with the five national unit standards and the critical reading section will include evidence-based medicine and primary care research methods.

This month’s edition is based on unit standard 1 (critically reviewing new evidence and applying the evidence in practice), unit standard 2 (evaluate and manage a patient according to the biopsychosocial approach), unit standard 3 (facilitate the health and quality of life of the family and community) and unit standard 4 (facilitate the learning of others regarding the discipline of family medicine, primary healthcare and other health-related matters). The theme for this edition is child health. We suggest that you attempt answering the questions (by yourself or with peers or supervisors), before finding the model answers online: http://www.safpj.co.za/.

Please visit the Colleges of Medicine website for guidelines on the Fellowship examination: https://www.cmsa.co.za/view_exam.aspx?QualificationID=9.

We are keen to hear about how this series is assisting registrars and their supervisors in preparing for the FCFP (SA) examination. Please email us your feedback and suggestions.

## Multiple choice question: Single best answer

A 19-year-old woman is referred to your district hospital for kangaroo mother care (KMC) as her baby now weighs 1.2 kg. The mother is taught about breastfeeding and nursing and medical care was provided. The baby’s weight is now 1.8 kg. The most appropriate time to discharge this mother and baby pair is:

- Later, as the target weight of 2 kg has not been achieved.
- Now as the target weight of 1.8 kg has been achieved.
- Now, if the baby has shown consistent weight gain of 20 g/day.
- Now, if the KMC score is greater than 15.
- Now, if the KMC score is greater than 19.

*Answer*: (e)

Kangaroo mother care is an important intervention used to address the needs of stable low birth weight (LBW) infants. This intervention is included in the Strategic Plan for Maternal, Newborn, Child and Women’s Health and Nutrition for South Africa and is specifically designed for stable LBW infants. Such LBW infants are often referred from regional or tertiary hospitals to district hospitals when the appropriate infrastructure and healthcare personnel are in place. This includes an adequate number of appropriately trained nurses and doctors. Complications from preterm birth account for 35% of neonatal deaths globally, so institutional KMC is regarded as an important intervention in dealing with the burden of this disease. Data from the Perinatal Problem Identification Programme (PPIP) showed that public hospitals implementing KMC were able to reduce their LBW mortality by 30%. The Department of Health (DoH) provides guidelines for the care of all newborns in district hospitals, health centres and midwife obstetric units in South Africa and most of the ensuing discussion could be found in this resource.

The mother kangaroo’s pouch provides warmth, safety and a constant supply of food (milk) to the joey and a similar analogy is used for the LBW infant and mother pair. The components of KMC include the following:

1. Kangaroo position: Skin-to-skin on the mother’s chest secured with a wrap.
2. Kangaroo nutrition: Exclusive breastfeeding whenever possible with nutritional supplementation.
3. Kangaroo monitoring.
  Kangaroo monitoring includes the following parameters:
  - Six-hourly vital signs (heart rate, respiratory rate, temperature), activity and colour.
  - Intake and output fluid monitoring.
  - Daily weight and the KMC score chart.
  - Weekly occipito-frontal head circumference and haemoglobin.
4. Kangaroo discharge: Mother continues the KMC practice at home after discharge.
5. Kangaroo support: Healthcare staff provides support to the mother to take care of her infant in the hospital.
  Kangaroo support includes:
  - Healthcare staff support in hospital
  - Emotional support – The mother needs encouragement if she is to provide KMC in a sustainable manner.
  - Teaching mothers the necessary skills so that they may effectively take care of their LBW infants.
  - After discharge infants need regular follow-up to check satisfactory weight gain at clinics close to home
  - Support from the family at home to help mother take care of her infant and practice KMC at home.
6. Family support of mother in practicing KMC at home.

**FIGURE 1:**
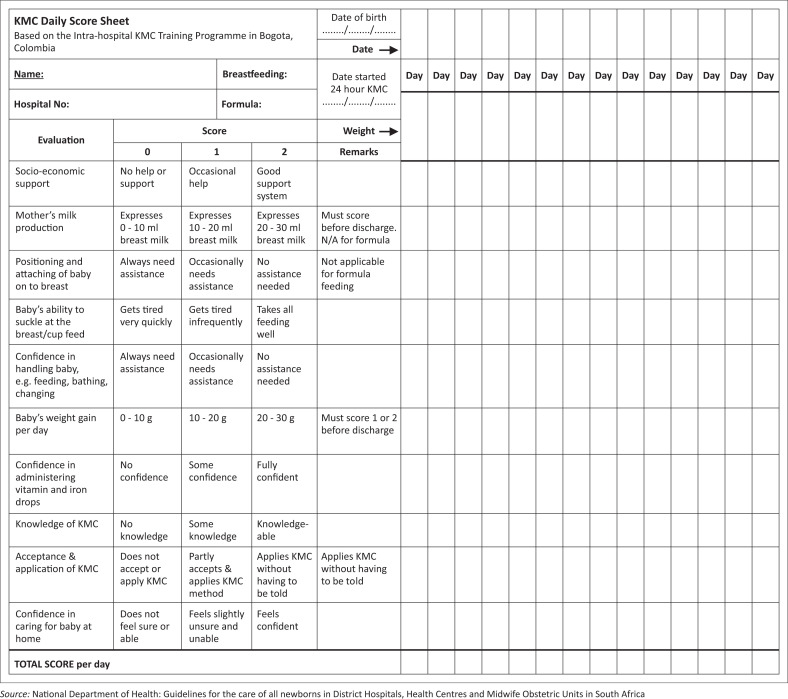
Kangaroo mother care daily score chart.

To ensure that the mother–baby pair continues to thrive, all 10 monitoring criteria must be achieved with a minimum score of 20 for breastfed babies and 16 for formula-fed babies. Figure 1 lists the criteria for the KMC score.

It is crucial that the mother–baby pair be managed holistically to ensure that the baby continues to gain weight once discharged from the district hospital and that the knowledgeable confident mother continues to receive socio-economic and emotional support, especially in the context of teenage mothers.

Further reading:

- South African Department of Health. Guidelines for the care of all newborns in district hospitals, health centres and midwife obstetric units in South Africa. Pretoria; 2014. Available from: http://www.kznhealth.gov.za/kinc/Newborn_care_charts_March_2014.pdf
- Feucht U, Van Rooyen E, Skhosana R, Bergh A-M. Taking kangaroo mother care forward in South Africa: The role of district clinical specialist teams. S Afr Med J. 2016;106(1):49–52. https://doi.org/10.7196/SAMJ.2016.v106i1.10149

## Short answer question: The family physician’s role as care provider

You are a family physician in the sub-district and support the development of community oriented primary care (COPC).

1. You are participating in a task team that is defining the scope of practice for community health workers (CHWs). From national policy and experience with COPC implementation, what key roles would you recommend for the CHWs to improve child health? (6 marks)
2. You are wondering about immunisation status and vitamin A administration in your community. A medical student has analysed data collected by the CHWs over the past 1 year. She presents the following results from 321 households, which include 1008 adults and children:
  - Interpret these findings. (3 marks)
  - Make appropriate recommendations based on these findings. (2 marks)
3. There seems to be a problem with CHW referrals to the clinic. Only 6 of the 28 children identified with not up-to-date immunisations were recorded as being referred on the Household Assessment form. What might be some possible reasons for this? (3 marks)
4. At the CHW team meeting, the local clinic sister reports that a number of children have been brought to the clinic dead, as a result of choking and asphyxiation. She believes that young mothers are trying to force their babies to eat solids before they are ready. As the family physician member of the team, how would you address this issue further? (6 marks)

**Total: 20 marks**

### Model answers

This question was used in a previous FCFP(SA) examination.

#### 1. You are participating in a task team that is defining the scope of practice for community health workers. From national policy and experience with community oriented primary care implementation, what key roles would you recommend for the community health workers to improve child health? (6 marks)

Community health workers can have key roles in the following areas:

- Identify people at risk during household visits, for example, sick children, newborns, those with mental health problems.
- Recognise and deal with minor ailments, for example, minor burns, nappy rash and diarrhoea.
- Promote health, for example, immunisations, encourage breastfeeding oral health and use of oral rehydration solution (ORS).
- Administer preventative measures such as deworming and giving Vitamin A.
- Screen for risk and disease, for example, check growth and development.
- Support adherence, for example, in the management of acute and chronic conditions, such as in children on HIV or tuberculosis (TB) medication.
- Identify those in need or physical care or rehabilitation, for example, cerebral palsy.
- Collect information on child health needs , for example, household assessment form and social determinants of health – housing, sanitation, safety, food, clean water and income.
- Contribute to community engagement, diagnosis and prioritisation of health needs for children, for example, participate in community health forum. Contribute to stakeholder engagement for child health, for example, identify assets in the community.
- Train Integrated Management of Childhood Illness (IMCI) red flags.
- Screen for child abuse/neglect.
- Provide support to parents or other adults caring for children – post-partum depression, HIV care for mother, etc.

#### 2. You are wondering about immunisation status and vitamin A administration in your community. A medical student has analysed data collected by the community health workers over the past 1 year. She presents the following results from 321 households, which includes 1008 adults and children (see Table 1)

- **Interpret these findings. (3 marks)**
*Interpretation (3 marks): Award points for the candidate’s understanding about the problems with the data set and therefore the limitations of conclusions that can be drawn, not just the analysis.*
  ■ This is a poor data set: information is lacking on most people so that interpretation is limited (do not award full marks if this is not commented on).
  ■ Of the *N* = 1008, we do not know who are children or adults.
  ■ People are not reflected within their households, so it is not possible to comment on particular ‘at risk’ households.
  ■ The denominator includes all people and not just the target group (children under 5 years) so the percentage appears lower than it really is.
  ■ If the percentages are recalculated out of those with a response recorded, then the percentage of those with immunisations not up to date increases to 16.8% (28/167) and for vitamin A 19.1% (31/162).
- **Make appropriate recommendations based on these findings. (2 marks)**
*Recommendations (2 marks): Award one mark for one reasonable recommendation that will improve data capture through training, more user-friendly tools, re-designing workflow and addressing the low uptakes of Vitamin A and immunisations.*
  ■ Explore with the CHWs and their supervisors about what is being asked in the process of data collection and how this is being recorded to ensure results are accurate and meaningful.
  ■ Completion of immunisation schedules and Vitamin A administration could be accomplished with a catch-up campaign, nursery school visits, etc.

**TABLE 1:** Immunisation and vitamin A administration (01 January–31 December 2019).

Child health indicator	Yes		No		No data available	
	*n*	%	*n*	%	*n*	%
Immunisations up to date	139	13.8	28	2.8	841	83.4
Vitamin A up to date	131	13.0	31	3.0	846	84.0

*Source:* Mash B, Du Pisanie L, Swart C, Van der Merwe E. Evaluation of household assessment data collected by community health workers in Cape Town, South Africa (submitted to peer-reviewed journal).

*N* = 1008 adults and children.

#### 3. There seems to be a problem with community health worker referrals to the clinic. Only 6 of the 28 children identified with immunisations not up to date were recorded as being referred on the Household Assessment form. What might be some possible reasons for this? (3 marks)

*Possible issues to consider regarding no record of children being referred to the clinic by CHWs (award 1 mark per distinct issue):*

- Systems issues: CHWs are referring children, but the information system needs revising or upgrading because the referrals are not being captured properly after the CHW does her job. Community health workers may not be referring because of ongoing pay or labour disputes with provincial departments of health.
- Healthcare worker issues: CHWs not sure whom to refer to because of lack of training. Community health workers are referring, but not recording or using incorrect forms. Problems within the teams – relationship with team leader, low involvement from family physicians or MOs providing leadership and training for the team.
- Patient issues: CHWs are reluctant to refer because of problems with the community in accessing the local clinic (lack of funds for transport, inaccessible hours, treatment of migrants, stigma, etc.)

#### 4. At the community health worker team meeting, the local clinic sister reports that a number of children have been brought to the clinic dead, as a result of choking and asphyxiation. She believes that young mothers are trying to force their babies to eat solids before they are ready. As the family physician member of the team, how would you address this issue further? (6 marks)

*As a family physician, what is your approach? Answer should exhibit features of leadership and clinical governance (6 marks):*

- Establish the facts by obtaining data (e.g., by conducting a base line audit) – Explore what really happened to the children who died (any reasonable suggestions), maybe the sister’s interpretation of the cause of death is not correct? For example, try and follow up with the clinic sister, the medical records and any post-mortem findings/inquest findings/M&M on the cases.
- Discuss with and involve stakeholders – name possible stakeholders including community leaders regarding infant feeding practices. Gender and ages of children. How were cases reported? Assess population.
- Explore CHW awareness of force-feeding problem. For example, discuss with the CHWs if they are aware of this issue in the community and whether they see it as a real concern? Have they been able to identify mother–babies at risk – born to inexperienced mothers who are unsupported or even alienated in the community?
- Design an intervention, activity and plan of action: *provide any three sensible suggestions*.
- Design intervention with team (depending on problem), such as awareness raising or capacity building; involve community and possibly non-governmental organisations (NGOs) where necessary.
- Discuss with the CHWs if they can offer extra support to these mothers and grandmothers in infant feeding and parenting and whether more widespread education is needed in the community? Are there any other groups working in the community that could help with the issue?

Further reading:

- Marcus T. A practical guide to doing COPC. Pretoria: Minuteman Press; 2015.
- Marcus T, Hugo J. Community-orientated primary care. In Mash B, editor. Handbook of family medicine. 4th ed. Cape Town: Oxford University Press, 2017; 334–359.

## Critical appraisal of quantitative research

Read the accompanying article carefully and then answer the following questions (*total 30 marks*). As far as possible use your own words. Do not copy out chunks from the article. Be guided by the allocation of marks with respect to the length of your responses.

Koetaan D, Smith A, Liebenberg A, et al. The prevalence of underweight in children aged 5 years and younger attending primary health care clinics in the Mangaung area, Free State. Afr J Prim Health Care Fam Med. 2018;10(1):1–5. Available from: https://phcfm.org/index.php/phcfm/article/view/1476.

1. What were the author’s key points in their argument for the social value of the study? (2 marks)
2. What were the authors’ key points in their argument for the scientific value of the research? (2 marks)
3. Comment critically on the sample selected for this study and any source of selection bias. (4 marks)
4. Comment critically on the sample size. (3 marks)
5. Critically appraise how well the authors describe the validity of the study instrument identified for use in this study. (3 marks)
6. The authors reported that the median gestational age was 39 weeks (IQR 23, 42 weeks). What does the median and inter quartile range (IQR) mean and how would you interpret this information? (3 marks)
7. Comment critically on how the HIV status and TB status of the children is reported. (4 marks)
8. Based on this study, give two reasons why this study might not have been able to show any association between HIV status and malnutrition. (2 marks)
9. Discuss the practical implications of these reported HIV results. Do you agree with the comments in the discussion? (2 marks)
10. What other limitation(s) of the study could you identify? (2 marks)
11. What could be the take home message? (3 marks)

### Model answers

#### **1. What were the author’s key points in his argument for the social value of the study?** (2 marks)

*Answer: Any 4 of the following points:*

- Freedom from hunger and malnutrition is a basic human right. However, malnutrition remains a huge problem. (1 mark)
- Malnutrition has been included in the Millennium Development Goals (MDG) to aim to reduce the mortality rate of children under 5 by two thirds. (1 mark)
- South Africa (SA) has a significant number of children under 9 years of age who are underweight and stunted. (1 mark)
- Between 2009 and 2013 all provinces in SA except for the Free State were able to reduce the incidence of severe malnutrition. (1 mark)
- In 2011 the Free State had the highest under 5 mortality in SA (72.1/1000 live births vs. 38.5/1000 live births). (1 mark)
- Fifty percent of all child deaths are associated with malnutrition. (1 mark)
- There is an association between poverty and well-being with poverty being both the cause and consequence of malnutrition. (1 mark)

#### **2. What were the authors’ key points in their argument for the scientific value of the research?** (2 marks)

The scientific value of a study relates to the knowledge gap. Here the authors highlighted the social value of the study (*why and how relevant or important is it to health needs in my context?*), without making the scientific value of the study explicit (*how will this study contribute to new knowledge?*). (1 mark)

From the study aim below, one may infer that the scientific knowledge gap appeared to revolve around the need to understand the prevalence and possible causes for the local context. The authors did not make this argument explicit in the introduction section. (1 mark)

Study aim:

- To determine the prevalence of underweight in children under the age of 5 years attending the primary health care (PHC) clinics in the Manguang area.
- To determine the possible underlying causes.

#### 3. Comment critically on the sample selected in this study and any source of selection bias. (4 marks)

- Six out of twenty-three clinics were chosen purposefully (although no criteria are given for how they were purposefully chosen) for participation in the study. This is essentially a convenient sample of clinics and does not necessarily reflect the busiest clinics or those with the most malnutrition. It may or may not represent an even spread of the clinics in the area. In addition, convenient days were selected to visit the clinics. It is not clear if there was a target for each clinic based on a percentage of the number of children attending. (1 mark)
- This was a convenient sample of those children who happened to be visiting on the day of the visits. It is not clear from the description why the children were attending the clinic. Were they attending because they were sick or were they attending for routine immunisation and follow-up? All that is stated is that consecutive patients presenting at the clinic were taken on the days when the researcher was at the clinic. (1 mark)

Sources of selection bias: (any 2 of the following points)

■ This study includes a narrow focus (clinic based), rather than a broad focus (general population or community based) sample when considering prevalence of malnutrition in this setting. This brings the representativeness or generalisability of the study into question in the broader population. (1 mark)
■ Children presenting without a road to health card were excluded from this study. These children might be most at risk of malnutrition. (1 mark)
■ There may be a difference between those children who are presenting because they are sick and those who are attending for routine growth monitoring and immunisation. (1 mark)

#### 4. Comment critically on the sample size. (3 marks)

- The authors state that the sample size was set at 240 for statistical and logistical considerations with no justification presented in the paper. (1 mark)
- In the limitations section, it states that the number of children who attended the clinic was less than expected, forcing the research team to abandon any form of random sampling and reduce the sample size – suggesting that they had started with a sample size, which was calculated. (1 mark)
- Ideally one should have an estimation of the population under study (under the age of 5 years attending PHC clinics), an estimated prevalence of malnutrition (given as 72.1/1000 live births), a precision (often used as 0.05) and a confidence interval (usually 95%). (1 mark)

#### 5. Critically appraise how well the authors describe the validity of the study instrument identified for use in this study. (3 marks)

- A valid questionnaire measures what it claims to measure. In this study, data on several variables were collected from the road to health chart. A data collection tool was piloted using three road to health charts from the six clinics where the data were to be collected from (18 charts in total). (1 mark)
- However, in the limitations section, the authors state that the mid-upper arm circumference (MUAC), length and head circumference were very rarely recorded and that the pilot study was too small to pick this up. (1 mark)
- There was an assumption that the data (weight and height) in the road to health charts were accurate and no attempt was made to confirm this (although it would have been possible to confirm some of the data when the children were seen at the clinic). This means that the anthropometric data gathered were not valid nor reliable, as the researchers did not check whether these measures where taken consistently across the study sample. (1 mark)

#### **6. The authors reported that the median gestational age was 39 weeks (IQR 23, 42 weeks). What does the median and inter quartile range mean and how would you interpret this information?** (3 marks)

- The *median* is the middle number in a sorted list of numbers (a median is used in continuous data that are not normally distributed). (1 mark)
- The *interquartile range* (IQR) is a measure of statistical dispersion and describes the difference between the 75th and 25th percentiles, or between upper and lower quartiles. (1 mark)
- Interpretation: According to this statement (*median gestational age was 39 weeks* [*IQR 23, 42 weeks*]), 50% of the children in this study were born between 23 weeks and 42 weeks with the middle number being 39 weeks. (1 mark)

#### 7. Comment critically on how the human immunodeficiency virus status and tuberculosis status of the children is reported. (4 marks)

- There is inconsistency in reporting of the data for HIV. In Figure 1, 18 children are reported to be HIV-positive, whilst in the text 21 children tested HIV-negative. In addition, in the text the percentage of children testing positive is given as 8.7%, which assumes that the denominator is 240 (21/240 = 8.75%). Whilst in Figure 1 the denominator is 222 (18/222 – 8.1%), suggesting that some of those reported in the text actually fall into the unknown or missing category. (1 mark)
- The authors state that most of the 240 children tested negative for TB. It is not clear who were tested or why they were tested. The study was conducted at a PHC clinic where the children presented. It is not clear if the children were sick or whether they were presenting at a well-baby clinic for immunisation and follow-up. If they were presenting at a well-baby clinic there would be no reason for testing for TB. In addition, it is difficult to test children under 5 years of age for TB (the median age for the children in this study was 7.5 months) and it is not clear how these children were assessed for TB. (1 mark)
- Based on the data given, there was no statistical association between the child being HIV-positive and the chance of presenting with malnutrition (*p* = 0.7217). Other studies have shown that the risk for malnutrition is significantly higher in HIV-infected children than in HIV-uninfected children. (1 mark)

#### 8. Based on this study, give two reasons why this study might not have been able to show any association between human immunodeficiency virus status and malnutrition. (2 marks)

*Any two of the following:*

- The sample size was too small. (1 mark)
- There really was no association between the groups in this study. (1 mark)
- Inaccurate measurements of height and weight may have hidden any difference between the groups. (1 mark)
- Most children in this study (93.2%) were breastfed for the first 3-months and were assessed at 7.5 months. There might only be a small actual difference between the two groups and the assumptions used for calculating the effect size were inaccurate. (1 mark)

#### 9. Discuss the practical implications of these reported human immunodeficiency virus results. Do you agree with the comments in the discussion? (2 marks)

- It is disturbing that 21 children tested positive for HIV (or 18 tested positive according to Figure 1) and that HIV results were unknown for 54 mothers and not recorded (unknown) for 72 children (64 according to Figure 1). (1 mark)
- The discussion suggests that HIV-testing should be done routinely in all children attending PHC clinic. Whilst this is true, the lack of results in the road to health chart suggest a breakdown of the prevention of mother-to-child transmission (PMTCT) programme. All mothers should be tested during antenatal care – initially when they first present and again at 32-weeks and again when they present in labour. Human immunodeficiency virus results should be recorded in the road to health chart for all children. (1 mark)

#### 10. What other limitation(s) of the study could you identify? (2 marks)

*Any two of the following:*

- This was a convenient sample and may not accurately represent the study population.
- The study population is not known and the sample size may not be suitable for statistical analysis.
- An assumption was made that measurements were accurate.

#### 11. What could be the take home message? (3 marks)

*Any three of the following:*

- Between 4.8% and 11.9% of children presenting to six PHC clinics in the Free State where malnourished. (1 mark)
- In total, 8.7% (or 8.1% – 18/222) of the children were HIV-positive. (1 mark)
- In total, 30% (or 28.8% – 64/222) did not have a HIV result in their Road to Health Cards – suggesting (1) possible breakdown in the PMTCT program, which needs to be investigated and (2) that the prevalence of those who are HIV-positive could be much greater than 8.7%. (1 mark)
- Anthropological measurements are poorly completed making it difficult to comment on stunting and to quantify the problem. (1 mark)
- There is a need to repeat the study with an adequate sample size and appropriate sample selection to confirm these findings. (1 mark)

**(Total: 30 marks)**

Further reading:

- Mash B, Ogunbanjo GA. African primary care research: Quantitative analysis and presentation of results. Afr J Prim Health Care Fam Med. 2014;6(1):1–5. https://doi.org/10.4102/phcfm.v6i1.646
- Mash B. African primary care research: Choosing a topic and developing a proposal. Afr J Prim Health Care Fam Med. 2014;6(1):1–6. https://doi.org/10.4102/phcfm.v6i1.580
- Govender I, Mabuza LH, Ogunbanjo GA, Mash B. African primary care research: Performing surveys using questionnaires. Afr J Prim Health Care Fam Med. 2014;6(1):1–7. https://doi.org/10.4102/phcfm.v6i1.589
- Pather M. Evidence-based family medicine. In: Mash B, editor. Handbook of family medicine. 4th ed. Cape Town: Oxford University Press, 2017; 430–453.
- Joannabriggs.org. Critical appraisal tools – JBI [homepage on the Internet]. 2019 [cited 2019 Aug 19]. Available from: http://joannabriggs.org/research/critical-appraisal-tools.html
- CASP Checklists. Critical Appraisal Skills Programme [homepage on the Internet]. c2018 [cited 2020 Aug 07]. Available from: https://casp-uk.net/casp-tools-checklists/

## Objectively Structured Clinical Examination scenario

### Objective of station

This station tests the candidate’s ability to:

- Engage in a supervisory discussion with a junior colleague.
- Manage (diagnose and treat) a paediatric patient with bacterial meningitis and underlying chronic malnutrition.

### Type of station

Integrated consultation – clinical management, complex consultation.

#### Equipment list

- Role player: male/female – adult
- Clinical findings – printed sheet
- Laboratory findings – blood and cerebrospinal fluid (CSF)
- Chest x-ray – printed
- Growth monitoring chart showing stunted growth

### Instructions for candidate

#### History/context

You are the family physician working in a district hospital.

The medical officer working in the paediatric ward asks for your opinion on one of the inpatients.

### Your task

- Engage with the doctor around the issues being raised
- Negotiate a management plan appropriate to your assessment

N.B. You do not need to perform a physical examination.

Physical examination findings and investigations will be provided to you on request.

### Instructions for the examiner

**Objectives:** This consultation station tests the candidate’s ability to:

- Engage in a supervisory discussion with a junior colleague
- Manage (diagnose and treat) a paediatric patient with bacterial meningitis and underlying chronic malnutrition

This is an integrated consultation station in which the candidate has 14 min.

**FIGURE 2:**
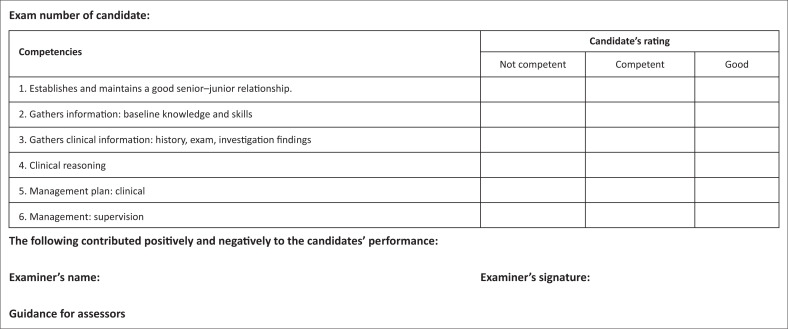
Marking template for consultation station.

No marks are allocated. In the mark sheet (see Figure 2), tick off one of the three responses for each of the competencies listed. Make sure you are clear on what the criteria are for judging a candidates’ competency in each area.

Provide the following information to the candidate when requested:

- Examination findings
- Laboratory findings CSF

Please switch off your cell phone.

Please *do not* prompt the student.

Please ensure that the station remains tidy and is reset between candidates.

This station is 15 minutes long. The candidate has 14 minutes, then you have 1 minute between candidates to complete the mark sheet and prepare the station.

**Reference:** Department of Health. Meningitis, Acute Bacterial. Essential Medicines List, Hospital Level, Paediatrics. 2017;239–241.

### Guidance to assessors

**FIGURE 3:**
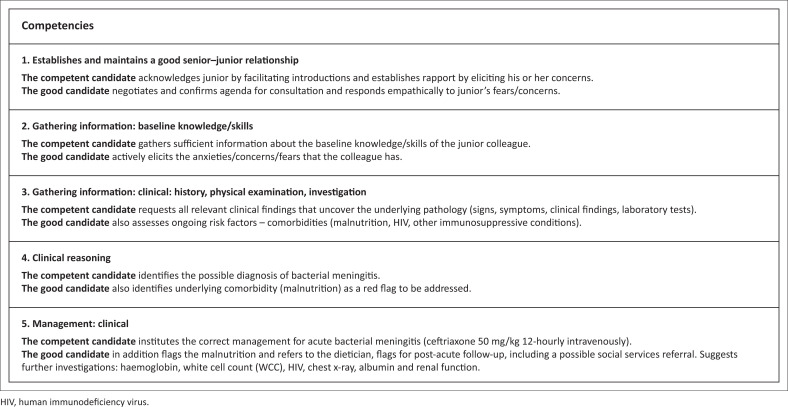
Guidelines for examiners to judge student competencies.

Familiarise yourself with the assessor guidelines (Figure 3), which details the required responses expected from the candidate.

The aim is to establish that the candidate can safely and effectively diagnose and manage a child with bacterial meningitis, whilst supervising a junior colleague in the process.

Competent: The task is completed safely and effectively.

Good: In addition to displaying competence, the task is completed efficiently and in an empathic, person-centred manner (acknowledges and responds to ideas, beliefs, expectations and concerns or fears).

### Role play – Instructions for actor

#### Appearance (including dress) and behaviour (emotions and actions)

You are a community service medical officer. You have just started your paediatric allocation and have not yet managed a child with meningitis. You need the senior doctor to review your diagnosis and management plan.

**Opening statement:** (Responds to doctor). ‘I think this child may have viral meningitis, but I’m not sure, and would like your opinion please’.

### History

#### Open responses: Freely tell the doctor if asked …

The child was admitted from the emergency unit last night, with a history of high fever and vomiting for 1 day. There was no loss of consciousness, and the doctor on duty did a lumbar puncture (LP), administered the first dose of ceftriaxone 1000 mg intravenously and admitted the child to the ward for ongoing management. He thought it was likely a viral meningitis.

This is the first time you are seeing this child.

#### Closed responses: Only tell the doctor if s/he brings this up

Your knowledge/skills/confidence in paediatrics is not great – you did your internship at a tertiary hospital where you saw mostly cardiology and endocrine patients. Working in the paediatrics ward is very stressful for you.

### Patient’s notes from emergency centre

***Four-year-old boy, brought by mother, with history of sudden onset fever and vomiting for 1 day***.

***Previously healthy, although day hospital referral says that he is receiving food supplements for moderate malnutrition.***

No history of TB contacts nor HIV exposure.

**On admission:** body mass 12 kilograms; temperature 38.9 °C; heart rate – 123/min; respiratory rate 24/min; random glucose – 6.7 mmol/L.

This morning: temperature 38 °C; heart rate 125/min; respiratory rate 22/min.

### Examination findings

- Thin child, slightly lethargic, but awake.
- Hot to touch.
- No generalised lymphadenopathy, mild pallor.
- Chest: good air entry bilaterally.
- Neuro: significant (+++) neck stiffness; no focal neurology; pupils equal and reactive.

### Assessment

meningitis, likely viral.

### Plan

- Drip up.
- LP, bloods – WCC.
- Start dose of ceftriaxone 1 g intravenously.
- Admit to the ward.

### Laboratory results

**Bloods:**

- WCC: 16.4

**CSF:** Appearance: *Turbid*

- Glucose: < 0.6 mmol/L
  ■ Protein: 4.41 g/L, (0.15–0.4)
  ■ Cell count:
  ■ Polymorphs: 900/*µ*L
  ■ Lymphocytes: 150/*µ*L
  ■ Erythrocytes: 20/*µ*L
  ■ Unidentified cells: 0
- Microscopy: Gram positive diplococci.
- Culture and sensitivity: pending.

